# Finger Sequence Learning in Adults Who Stutter

**DOI:** 10.3389/fpsyg.2020.01543

**Published:** 2020-07-24

**Authors:** Alexandra Korzeczek, Joana Cholin, Annett Jorschick, Manuel Hewitt, Martin Sommer

**Affiliations:** ^1^Department of Clinical Neurophysiology, University Medical Center Göttingen, Göttingen, Germany; ^2^Faculty of Linguistics and Literary Studies, Bielefeld University, Bielefeld, Germany

**Keywords:** stuttering, motor sequence learning, finger tapping, overnight consolidation, generalization, adults, speed, accuracy

## Abstract

Originary neurogenic, non-syndromatic stuttering has been linked to a dysfunctional sensorimotor system. Studies have demonstrated that adults who stutter (AWS) perform poorly at speech and finger motor tasks and learning (e.g., Smits-Bandstra et al., 2006b; Namasivayam and van Lieshout, 2008). The high relapse rate after stuttering treatment could be a further hint for deficient motor learning and, in particular, for the limited generalization of the learned technique in daily communication. In this study, we tested generalization of finger sequence skills in AWS using an effector-dependent transfer task after a 24-h retention period. Additionally, we wanted to corroborate previous motor learning results in AWS for practice and retention: 16 AWS and 16 age-, sex-, and education-matched controls performed the task during four test sessions. Our results indicate that generalization performance in AWS was not inferior to that of fluent controls. In addition, we found, contrary to previous results, that AWS showed a steeper learning progress after practice and consolidation compared with controls. We suggest that with sufficient practice and a 24-h consolidation phase, AWS are able to retain the learned performance of tapping a five-item finger sequence as well as fluent controls in terms of speed and accuracy.

## Introduction

Originary neurogenic, non-syndromic stuttering is a speech fluency disorder characterized by involuntary speech fluency disruptions (Neumann et al., 2016). Originary stuttering in childhood fortunately has a high spontaneous recovery rate of up to 80%. In those individuals in whom stuttering persists into adulthood, stuttering treatment is characterized by a high relapse rate even after intensive therapy (Craig, 1998). It is conceivable that the unifying trait differentiating persons with persistent stuttering from recovered individuals could be limitations in the motor learning of speech skill (Zelaznik et al., 1997; Peters et al., 2000; Max et al., 2004). As speech represents the skilled sequential organization of distinct, timed movement units in a pre-specified order (Schmidt and Wrisberg, 2008), motor sequence learning may play a central role in speech skill development. Thus, limitations in the speech skill of stuttering individuals could emerge due to the limited ability to learn motor sequences. In line with this suggestion, studies have reported poorer speech sequence skill learning in adults who stutter (AWS) than in those who do not (ANS; Smits-Bandstra et al., 2006b; Namasivayam and van Lieshout, 2008; Smits-Bandstra and De Nil, 2009; Smith et al., 2010; Bauerly and De Nil, 2011; Smits-Bandstra and Gracco, 2013, 2015; Sasisekaran and Weisberg, 2014). Other studies have used finger tapping tasks to investigate if limitations in motor sequence learning or performance also affect non-speech movements in AWS (Webster, 1986; Smits-Bandstra et al., 2006a, b; Bauerly and De Nil, 2015).

Generalization describes the ability to transfer the learned motor performance on similar but not practiced movements (Schmidt and Wrisberg, 2008; Witt et al., 2010). To our knowledge, only one study has tested generalization of speech and finger motor sequence learning in AWS (Smits-Bandstra et al., 2006b). In their study, the finger tapping transfer task was conducted on the same day as the original motor sequence task and consisted of a new sequence. For both modalities, speech and finger tapping, ANS transferred the improvements in reaction time of the practiced movements faster than AWS (Smits-Bandstra et al., 2006b). These results suggest that AWS might have difficulties in speech as well as non-speech motor sequence learning.

### Primary Objective

Our primary intention was to investigate finger motor sequence learning in AWS and ANS at different time intervals incorporating the effect of sleep on retention and on generalization. The finger tapping task that we used in the current study might reveal general limitations in motor sequence learning in AWS (Bauerly and De Nil, 2015). Several studies have investigated retention effects after 24 h in speech motor sequence learning tasks (Namasivayam and van Lieshout, 2008; Bauerly and De Nil, 2011; Sasisekaran and Weisberg, 2014), but only one studied finger motor sequence learning (Bauerly and De Nil, 2015). So far, generalization in AWS has been investigated only during a 1-day period, i.e., disregarding sleep effects on consolidation (Smits-Bandstra et al., 2006b). The current study implemented the dependent variable “triplet errors” (TEs) as a measure of accuracy. TEs are a fine-grained analysis of error type and may reflect the stability of sequence representation (Albouy et al., 2012). TE can occur within or between sequences. The authors proposed that an increase of within-sequence TE might represent increasing variability of motor sequence execution.

We hypothesize (1) that AWS will show limitations in motor sequence learning (lower increase in movement speed and accuracy between testing sessions) and (2) that AWS will perform more poorly than ANS at each test session with regard to speed and for accuracy. With an additional analysis of error type, we expect (3) that AWS will show more TE within a sequence, indicating greater variability of sequence execution than ANS (Albouy et al., 2012).

## Materials and Methods

### Participants

The Ethics Committee of the University Medical Center Göttingen approved this study, and written informed consent was obtained from all participants. Sixteen participants per group were matched for sex, age, education, and musicality (Table 1). Participants with professions requiring profound hand motor skills (e.g., computer scientist) were equally often present in both groups. All participants were right handed according to the Edinburgh Handedness Inventory (Oldfield, 1971), and all had normal hearing acuity (whispered voice test, MacPhee et al., 1988). In the AWS, stuttering severity (Stuttering Severity Index, Riley, 2009) ranged from mild to severe: six very mild, three mild, five moderate, and two severe. Control participants did not show any signs of speech dysfluency. Participants declared they had no pre-existing neurological condition or restricted movement of the fingers or hands, nor did they use drugs or medication that influence the central nervous system. The AWS were recruited through the Kasseler Stottertherapie and self-help groups in Bielefeld, Dortmund, Göttingen, Hannover, Münster, and Würzburg. ANS were contacted via local advertisement and at the Bielefeld University Campus.

**TABLE 1:** Descriptive statistics of demographic information and pretest results.

	AWS	ANS	Test statistics
Number of participants (female)	16 (2)	16 (2)	–
Age (years)	33 (12.6)	31 (10.7)	*t*(29.2) = −0.37, *p* = 0.715
Education, S-2C/HS-2C/HS-4C (*n*)	3/4/9	3/4/9	No test necessary, as groups were pairwise matched based on education
Musicality—plays instrument regularly/occasionally/never (*n*)	3/1/12	1/2/13	χ^2^(2) = 1.37, *p* = 0.5
**Pretests**			
DSF, mean score (SD)	9.94 (1.98)	10.06 (2.24)	*t*(29.6) = 0.17, *p* = 0.868
DSB, mean score (SD)	8.75 (2.27)	9.19 (1.87)	*t*(28.9) = 0.59, *p* = 0.556
2-B V, median percentage correct answers	58.57	85.00	*W* = 162.5, *p* = 0.189
2-B A, median percentage correct answers	83.33	83.33	*W* = 119.5, *p* = 0.745
SRT, mean ms (SD)	298.51 (20.46)	309.26 (26.93)	*t*(27.9) = 1.27, *p* = 0.214
CRT, mean ms (SD)	471.78 (58.52)	466.99 (40.48)	*t*(26.6) = −0.27, *p* = 0.79

*Total counts, medians or means, and standard deviations are given for each group (AWS, adults who stutter; ANS, adults who do not stutter). Education is categorized by school and college graduation (S-2C, school and 2 years of college; HS-2C, high school and 2 years of college; HS-4C, high school and 4 years of college). Tests of working memory (DSF, digit span forward; DSB, digit span backward; 2-BV, 2-Back Visual; and 2-BA, 2-Back Auditory) and reaction time (SRT, single reaction time; CRT, choice reaction time). The test statistics are from two-sided unpaired *t* tests for age, DSF, DSB, SRT, and CRT, Pearson chi-square for education and musicality, and unpaired Wilcox rank-sum test tests for 2-BV and 2-BA.*

### Pretests

Working memory can influence outcome measures in sequence skill learning (Seidler et al., 2012). Two preliminary tests of working memory were therefore administered to ensure comparability between the groups. Working memory was tested using the Digit-Span forward and backward subtests of the Wechsler Adult Intelligence Scale test battery (WAIS; Petermann and Wechsler, 2012), and the visual and auditory 2-Back test (Brain workshop Version 4.8.8). In the WAIS Digit-Span subtests, participants immediately repeated auditorily presented numbers either forward or backward. For the auditory and visual 2-Back task, participants were required to indicate whether the current item was the same as the item presented two trials previously. In addition, we assessed inter-individual differences in response latencies between participants using the Deary–Liewald single and choice reaction time task (Deary et al., 2011). In the single reaction time task, participants had to respond as quickly as possible to the appearance of a single black stimulus (“X”) by pressing a key. The black stimulus appeared 21 times in a white square, which was located in the middle of a blue screen. The choice reaction time task consisted of four white squares in one horizontal line across the middle of a computer screen. The black stimulus would appear randomly 40 times in any of these four white squares. Each square had a corresponding key on the keyboard. With the appearance of the black stimulus in one of the squares, participants had to press the corresponding key as quickly as possible (Deary et al., 2011). A random interstimulus interval prior to the following stimulus (1.0, 2.0, or 3.0 s) minimized anticipation effects on single and choice reaction time. Groups did not differ significantly in any pretest (Table 1).

### Motor Learning Task

**FIGURE 1:**
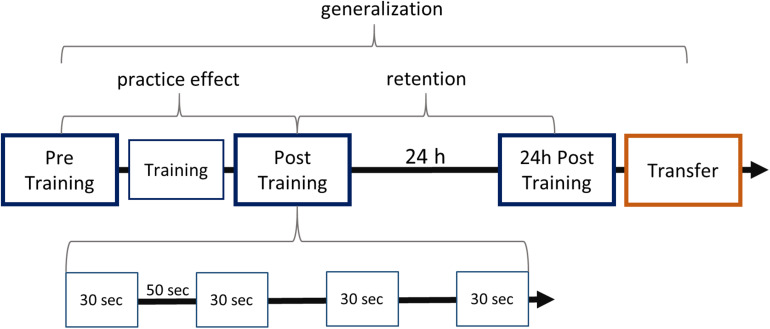
Study design and learning paradigm. The experiment consisted of five modules, which are aligned on the first arrow. The arrow represents the progress in time, marking the 24-h break including sleep between the first two and the last two sessions. Blue boxes stand for sequence tapping with the left hand and are named Pre Training, Post Training, 24-h Post Training. The red box represents the Transfer task, which was performed with the right hand. Training consisted of typing the sequence 160 times after a start stimulus. Testing sessions each contained four 30-s blocks separated by 50-s intervals as shown for Post Training. Practice effects were defined by the comparison between Post Training and Pre Training. Retention was studied by comparing performance at 24-h Post Training and at Post Training. Generalization was defined as the improved performance at Transfer compared with performance at Pre Training.

We used a finger tapping task to test motor sequence learning in a group of AWS and a group of ANS. Motor sequence learning was defined as the change in performance between four testing sessions: Pre Training, Post Training, 24-h Post Training, and 24-h Transfer. The testing sessions consisted of four blocks, each lasting for 30 s of tapping the pre-given sequence. Each block began with an auditory start signal (two consecutive 400-Hz tones lasting 2.5 s) and ended with a short display of a written word (“Pause”). Blocks were separated by 50 s (Figure 1), to give the participants enough time to rest (Korman et al., 2003). During the experiment, the sequence was not displayed on the screen. A training session of 160 repetitions was conducted between Pre Training and Post Training (Korman et al., 2003). Unlike the four testing sessions, the training session required 160 cued responses of one sequence at a time. The cue stimulus was the same auditory start signal as in the testing session.

Prior to the finger tapping task, participants were given written instructions to tap in the introduced sequence (41324) as fast and as accurately as possible after an auditory start signal. The instruction resulted in iterations of the sequence “41324-41324-41324- and so on.” The instructions prompted the participants not to correct errors by going back to the last correct key in their sequence, but rather to continue the sequence. Participants tapped the sequence on a Microsoft Natural Ergonomic 4000 Keyboard, using the keys [x c v b] for the left hand and [m, . -] for the right hand. To exclude the possibility of tapping the wrong keys, all other keys were covered. The keyboard was concealed in a box to prevent the participants from receiving visual feedback.

The finger tapping task comprised sequence “41324,” performed as the sequence of little finger, index finger, ring finger, middle finger, and little finger. This sequence, as well as similar five-digit sequences, has been reported to effect motor sequence learning in adults (Korman et al., 2003; Fischer et al., 2005; Witt et al., 2010; Albouy et al., 2012). The study design is similar to that of Korman et al. (2003): Pre Training, Training, Post Training, and 24-h Post Training were completed using the non-dominant (left) hand. For the Transfer condition, participants completed the same finger sequence with the dominant (right) hand. Because the same fingers of the other hand were used, this condition is termed effector-dependent transfer of the sequence. The use of the same fingers of the other hand demands a spatially mirroring of the sequence (intrinsic transformation; Witt et al., 2010). Presentation software (Version 0.71) was used to control the experiment and record the results.

### Dependent Variables

We used three dependent variables to measure the improvement of motor performance, i.e., practice effect, retention, and generalization. We defined motor sequence learning as an improvement of movement speed and movement accuracy over time.

First, we assessed movement speed by the number of correct sequences (NCSs; Korman et al., 2003). As participants get faster, they can produce more sequences within a given time interval. To obtain the total NCS per testing session (Pre Training, Post Training, 24-h Post Training, or 24-h Transfer), all correct sequences of a given testing session were counted automatically in each block (30-s interval), resulting in four NCS values per participant and testing session. The additional analysis of early learning was conducted on these four values (one per block) per participant of the pre-training session only. If a sequence at the end of the 30-s interval was incomplete, the keystrokes were excluded from the analysis of speed.

To quantify accuracy, we calculated so-called TE as introduced by Albouy et al. (2012). The nature of TE allows a fine-grained analysis of error type (within and between errors) and is more suited to reveal strategic changes in sequence execution. For this analysis, all key taps of a participant during one block were treated as a long chain regardless of sequence correctness. A sliding window of three elements was then applied on this chain, block by block. This sliding window extracted all possible triplets. For example, for a correct sequence, 41324, the five possible correct Triplets were 413, 132, and 324 (within-sequence triplets) and 244 and 441 (between-sequence triplets; Albouy et al., 2012). Triplets deviating from the predefined triplets were counted as “triplet errors” (e.g., the incorrect sequence 4-41341244321324-4 contained seven within-TE: 134, 341, 412, 124, 432, 321, 213 and one between-TE: 443). Keystrokes (≥2) at the end of the 30 s, which did not correspond to the sequence or the correct triplet, were counted as an incorrect sequence or triplet and were added to the number of errors and TE.

A custom-written script (Perl version 5.16.3.1604) automatically implemented these procedures. No trials were excluded, since participants did not show any signs of distractions such as coughing during the 30 s of task execution.

### Statistical Procedures

To investigate the effects of practice, the performance after training (Post Training) was compared with that of Pre Training. Similarly, for the statistical analysis of retention, performance at 24-h Post Training was compared with performance at Post Training on the first day. As in Korman et al. (2003), generalization to the dominant hand was examined by comparing performance at Pre Training with 24-h Transfer (see Figure 1). To detect early learning differences, we compared the performance across the four blocks of Pre Training: Here, the differences between blocks indicate the learning progress of groups.

To analyze differences in speed (NCS) and accuracy (TE) between testing sessions and groups, we used non-parametric linear mixed-effects models (Pinheiro and Bates, 2000; Bates et al., 2015b). NCS was modeled under the assumption of a Poisson distribution. The distribution of all TE was negatively skewed. Therefore, the TEs were log transformed after adding a constant of 0.5. The log-transformed TE approximately followed a normal distribution, and the linear mixed-effect modeling was conducted accordingly. Generalized linear mixed-effects models are an extension of a Poisson regression that incorporates the effects of repeated measurements. Mixed-effects models have great advantages in dealing with unbalanced or non-normal data such as ours. The variance explained by main effects and interactions can be tested via likelihood ratio tests between successively reduced nested models (e.g., Baayen and Milin, 2010).

We tested the effects of Group (AWS and ANS) and Testing Session (Pre Training, Post Training, 24-h Post Training, and 24-h Transfer) on our dependent variables, NCS and TE. An additional predictor of TE was type of error. Two model fits were conducted for different subsets of data: (1) practice and retention (Pre Training, Post Training, and 24-h Post Training) and (2) generalization (Pre Training and 24-h Transfer). Group served as a between-subjects factor and Testing Session served as a within-subjects factor. Successive difference contrasts were used in the regression models for both Group and Testing Session comparisons. All models included the maximal random effects structure justified by the data, a procedure to reduce the random effect structure by means of model comparisons suggested by Bates et al. (2015a, 2018). Moreover, model comparisons via likelihood ratio tests were used to determine the *post hoc* statistics of the main effects and interactions. Confidence intervals were calculated using the profile method from the lme4 package (Bates et al., 2015b, Version 1.1-19).

In addition, we calculated correlations between working memory capacity (digit span backward, visual and auditory 2-back) and an early increase of motor performance during Pre Training (NCS at B4 - NCS at B1). As groups did not differ in working memory capacity, correlation tests were calculated across all participants. We used Pearson’s correlation for normally distributed data and Spearman’s correlations for skewed data distributions.

## Results

### Motor Learning

Two AWS and one ANS showed systematic errors during the 24-h Transfer. One AWS and one ANS did not perform the effector-dependent Transfer task (mirroring the sequence and moving the same fingers of the other hand) but applied an extrinsic, effector-independent transformation by typing consequently 14231 instead of 41324 (using other fingers but keeping the spatial coordinate frame of the sequence). Another AWS typed all sequences during the first Transfer block as 41234 instead of 41324. Neither the sequence nor the triplet approach of errors enabled us to interpret these systematic errors. Therefore, each dependent variable was analyzed (1) for effects of practice and retention (Pre Training, Post Training, and 24-h Post Training) with all participants included and (2) for effects of generalization (Pre Training and 24-h Transfer) without these three participants.

For an additional analysis excluding the outliers from Pre Training, Post Training, and 24-h Post Training, see Appendix.

#### NCS: Practice and Retention

The first generalized linear mixed-effects model involved NCS as the dependent variable, and Testing Session (Pre Training, Post Training, and 24-h Post Training) and Group (AWS and ANS) as predictors. Overall, 7167 correct sequences were included in the analysis. For detailed descriptive statistics, see Table 2.

**TABLE 2:** Descriptive statistics on number of correct sequences by group and testing session.

Group	Pre Training (all)	Post Training (all)	24-h post Training (all)	Pre Training (subset)	Transfer (subset)
**ANS**					
Median	14.5	19.0	20.0	14.0	18.0
Mean	15.5	19.9	20.7	15.6	18.5
SD	5.2	4.6	5.7	5.4	6.4
Sum of NCS	993.0	1271.0	1325.0	933.0	1112.0
**AWS**					
Median	14.0	19.0	21.0	14.0	19.0
Mean	14.1	19.9	21.9	14.7	19.8
SD	6.0	5.5	6.3	6.1	5.8
Sum of NCS	900.0	1274.0	1404.0	822.0	1107.0

*Medians, means, standard deviations, and total sum of correct sequences (NCS) by Group (AWS, adults who stutter; ANS, adults who do not stutter) and Testing session. All: *n* = 16 per group. Subset: AWS *n* = 14, ANS *n* = 15.*

**FIGURE 2:**
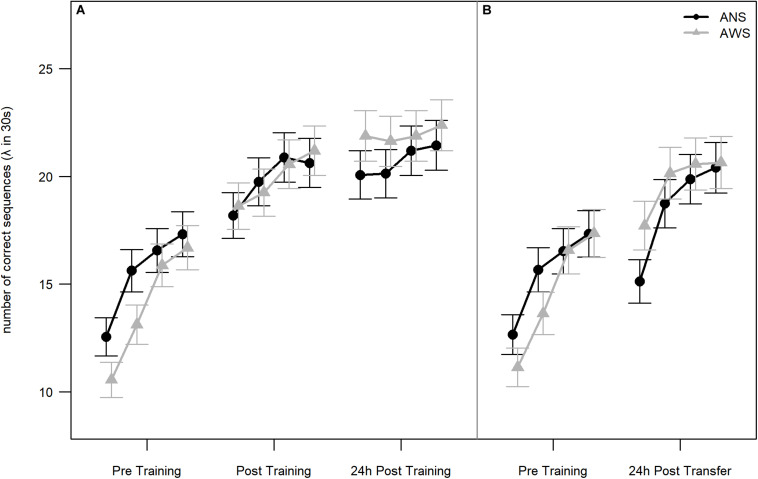
Number of correct sequences: Practice, retention, and generalization. Lambda of number of correct sequences (NCSs) is given for each block per test session. In Poisson distributions, lambda represents the mean occurrence per interval. Blocks are not part of the conducted analyses, but are visualized for additional information of the participants’ learning slopes. **(A)** Early learning, practice, and retention effects on NCS for adults who stutter (AWS; *n* = 16) and ANS (*n* = 16) [error bars represent the estimated standard errors using the formula sqrt(lambda(*x*))/sqrt(length(*x*))]. **(B)** Generalization effect on NCS for AWS (*n* = 14) and ANS (*n* = 15). The graph represents the analysis without the three outliers. The reasons for excluding these participants are described in section “Motor Learning” (error bars represent the estimated standard errors).

Both groups showed sequence motor learning, as implicated by a significant effect of Testing Session [χ^2^(2) = 167.2, *p* < 0.001]. The learning progress, i.e., difference between testing sessions, is reflected by the contrasts. The effect of practice was an approximately 30% increase in performance between Pre Training and Post Training [β = 0.3, SE = 0.03, 95% CI (0.24, 0.36)] on the first day. The effect of retention was a smaller but significant increase in performance after sleep of approximately 7% in the 24-h Post Training session compared to Post Training: β = 0.07, SE = 0.03, 95% CI (0.02, 0.12). There was no significant difference between groups: χ^2^(1) = 0.01, *p* = 0.919. Importantly, the interaction between Group and Testing Session was also significant [χ^2^(2) = 6.8, *p* = 0.033], indicating a difference in learning progress between groups. The significant interaction between Group and Testing Session results mainly from the change between the Pre Training and both Post Training sessions. AWS typed fewer correct sequences than ANS during the Pre Training session but caught up to the performance of ANS at Post Training and typed even more sequences than ANS at 24-h Post Training (see Figure 2A). None of these simpler interactions were significant by themselves [Group × Post Training - Pre Training: β = 0.1, SE = 0.06, 95% CI (−0.02, 0.22); Group × 24-h Post Training - Post Training: β = 0.06, SE = 0.06, 95% CI (−0.05, 0.16)].

#### NCS: Generalization

The Pre Training and Transfer sessions (see Table 2, the two rightmost columns, and Figure 2B) were compared on the subset of participants as described above. In total, 3974 correct sequences went into this analysis. We found a significant increase in performance in both groups [approximately 24% better performance in the Transfer than in the Pre Training session: χ^2^(1) = 54.3, *p* < 0.001]. Thus, participants in both groups were able to generalize the learned sequence to the other hand. However, there were no significant differences between groups [χ^2^(1) < 1, *p* = 0.879], and the interaction just missed significance [χ^2^(1) = 3.6, *p* = 0.056].

#### NCS: An Additional Analysis of Early Learning

To examine early learning in AWS and ANS, we conducted a further analysis of the 1893 correct sequences from the Pre Training session only. For detailed descriptive statistics, see Table 3. Using a Poisson distribution for modeling, we investigated the differences between Block and Group. Early learning was present in both, and we found a significant main effect for Block: χ^2^(3) = 39.1, *p* < 0.001 (see Figure 2A). There was a significant, 22% increase in the NCS between the first and the second block [β = 0.22, SE = 0.07, 95% CI (0.08, 0.36)]. The 12% increase between the second and the third block was also significant [β = 0.12, SE = 0.064, 95% CI (0, 0.25)]. There was no further significant increase between the third and the fourth blocks. The groups again did not differ significantly [χ^2^(1) = 0.9, *p* = 0.349]. In addition, the interaction between Block and Group was not significant: χ^2^(3) = 2.1, *p* = 0.552.

**TABLE 3:** Descriptive statistics on number of correct sequences per block during Pre Training.

Group	Block 1	Block 2	Block 3	Block 4
**ANS**				
Median	11.0	14.5	16.0	17.0
Mean	12.6	15.6	16.6	17.3
SD	5.0	5.4	5.1	4.6
Sum of NCS	201.0	250.0	265.0	277.0
**AWS**				
Median	9.5	12.0	15.5	15.5
Mean	10.6	13.1	15.9	16.7
SD	4.4	6.6	6.0	5.4
Sum of correct sequences	169.0	210.0	254.0	267.0

*Medians, means, standard deviations, and total sums of correct sequences (NCS) by group (AWS, adults who stutter; ANS, adults who do not stutter) for every block during Pre Training. *n* = 16 per group.*

Neither the digit span backward test (*r*_P_ = 0.06, *t* = 0.34, *p*_P_ = 0.73), nor the auditory *n*-back task (*r*_S_ = 0.14, *S* = 4,687, *p*_S_ = 0.44) or the visual *n*-back task (*r*_S_ = −0.05, *S* = 5,731, *p*_S_ = 0.78) correlated significantly with increase of NCS during Pre Training (Δ = B4 - B1).

#### Within-Sequence and Between-Sequence TE: An Analysis of Accuracy

##### TE: practice and retention

The participants produced 1232 TE in the first three sessions, 800 within-sequence errors, and 432 between-sequence errors (see Table 4 for descriptive statistics).

**TABLE 4:** Descriptive statistics on triplet errors by group and testing session.

		Pre Training		Post Training		24-h Post Training		Pre Training		Transfer	
Group	Estimate	Within	Betw.	Within	Betw.	Within	Betw.	Within	Betw.	Within	Betw.
ANS	Median TE	7.5	3.0	6.5	3.0	8.0	3.0	8.0	3.0	14.0	9.0
	*N* TE	129	67	223	76	129	69	122	64	227	146
	Mean TE.log	1.8	1.2	2.1	1.2	1.5	1.0	1.8	1.2	2.3	1.8
	SD TE.log	1.0	0.9	1.1	1.0	1.4	1.2	1.0	1.0	1.2	1.3
AWS	Median TE	2.5	2.0	3.5	3.0	2.5	1.5	2.0	1.5	3.5	2.0
	*N* TE	89	52	152	118	78	50	70	36	108	69
	Mean TE.log	1.0	0.8	1.5	1.4	0.8	0.7	0.8	0.7	1.5	1.2
	SD TE.log	1.4	1.1	1.5	1.3	1.4	1.2	1.4	1.1	1.2	1.1

*Medians and number (*N*) of TE and mean and standard deviations (SD) of the log-transformed triplet errors (TE.log) by Group (AWS, adults who stutter; ANS, adults who do not stutter) for Testing Session. All: *n* = 16 per group. Subset: AWS *n* = 14, ANS *n* = 15, betw., between.*

**FIGURE 3:**
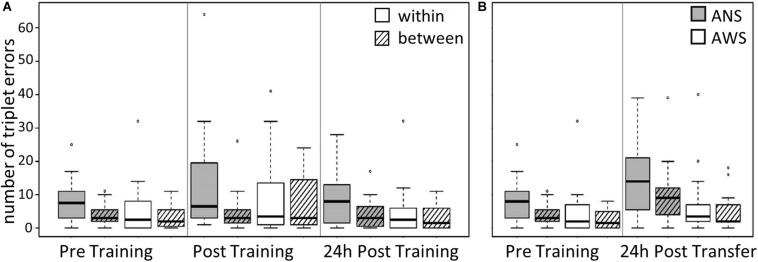
Triplet errors: Practice, retention, and generalization. The boxes outline the upper and lower quartiles of the median (black line). Whiskers represent the variability outside the upper and lower quartiles. Circles represent outliers. **(A)** Practice and retention differences of incorrect triplets (within vs. between) by Group (*n* = 16). ANS produce more within-sequence triplet errors (TE) than between-sequence errors compared with AWS (*p* = 0.012). **(B)** Generalization effect of incorrect triplets (within vs. between) for AWS (*n* = 14) and ANS (*n* = 15). ANS produce more TE than AWS (*p* = 0.031).

Group, Error Type, and Testing Session served as predictors. The same procedure as in the previous models was used to reduce the random effects structure. Interestingly, we found a two-way interaction between Group and Error Type [χ^2^(1) = 6.3, *p* = 0.012]: ANS made far more errors within a sequence than between sequences, while this difference was much smaller in the AWS Group [cf. Table 4, β = 0.51, SE = 0.2, 95% CI (0.12, 0.91)] (see Figure 3A). The main effect of Error Type also became significant, showing that, overall, participants made more errors within a sequence than between sequences [χ^2^(1) = 11.7, *p* < 0.001]. Moreover, sequence motor learning was expressed by the significant main effect for Testing Session [χ^2^(2) = 8, *p* = 0.018]. In terms of log-transformed TE, a trend showed that participants made slightly more within- and between-sequence TE after Training [β = 0.32, SE = 0.19, 95% CI (−0.07, 0.7)], which decreased again in the 24-h Post Training session [β = −0.52, SE = 0.2, 95% CI (−0.92, −0.11)] (Figure 3A). The main effect of Group did not become significant and none of the other interactions.

##### TE: generalization

In the subset without the three excluded participants used for the modeling of Pre Training and 24-h Transfer, the participants produced 842 TE: 527 within-sequence and 315 between-sequence (see the four rightmost columns of Table 4 for descriptive statistics).

The modeling was conducted as described above, using the log-transformed TE as the dependent variable and Group, Error Type, and Testing Session as predictors. The main effect of Error Type was significant [χ^2^(1) = 16.2, *p* < 0.001], showing that, overall, participants made fewer between-sequence TE than within-sequence errors [β = −0.37, SE = 0.08, 95% CI (−0.54, −0.2)]. The main effect of Testing Session was also significant [χ^2^(1) = 5.9, *p* = 0.015]: the participants made more TE during Transfer than during Pre Training [β = 0.57, SE = 0.22, 95% CI (0.12, 1.02)] (see Figure 3B). Moreover, we found a significant effect for Group [χ^2^(1) = 4.6, *p* = 0.031]; that is, AWS made fewer errors than ANS [β = −0.74, SE = 0.33, 95% CI (−1.41, −0.07)]. None of the interactions were significant.

## Discussion

We investigated motor sequence learning using a finger tapping task in a group of AWS and ANS. The study session included testing sessions after sleep. On the first day, the participants were tested before (Pre Training) and after (Post Training) a training session to investigate the effects of practice. On the second day, overnight consolidation (24-h Post Training) and generalization (24-h Transfer) to the dominant hand were assessed. Each testing session consisted of four blocks of 30 s each. Motor learning was interpreted as the gain in speed and accuracy due to practice and overnight sleep. Our results for speed and accuracy were unexpected in the context of the results of previous studies. We found that AWS were able to catch up quickly to the speed of ANS during a motor sequence-learning finger tapping task. Retention and transfer of the learned sequence after 24 h were similar in both groups.

### Movement Speed—Retention and Generalization

The finger tapping task elicited motor learning in both AWS and ANS after practice and consolidation in accordance with previous results (Korman et al., 2003; Fischer et al., 2005; Albouy et al., 2012, 2013). Thus, the participants were able to successfully generalize their speed-related performance due to practice to the non-dominant hand. The generalization effect is in line with the results in both transfer sessions (same day as training and 48 h post training) of Korman et al. (2003). Other studies that failed to find a generalization effect for effector-dependent transfer tasks (Thut et al., 1996; Witt et al., 2010) included neither spacing in the testing sessions nor an additional opportunity to practice prior to the Transfer Session. In the current experiment, each testing session included 50-s pauses between blocks. Also, on the second day, the Transfer task was scheduled after the block 24-h Post Training. The participants therefore practiced the sequence with the left hand one more time prior to Transfer. Both spacing during practice (Shea and Kohl, 1991) and delayed practice (Savion-Lemieux and Penhune, 2005) are known to increase motor sequence learning. Across all testing sessions, no main effect of Group was found, and movement speed in any testing session was similar between groups.

#### Stronger Motor Learning in AWS

The finding of a significant interaction between Group and Testing Session for the Pre Training and both Post Training sessions may suggest different motor sequence learning between our two groups. As can be seen in Figure 2, AWS started at a lower performance level than ANS, but at Post Training, AWS caught up to the performance level of ANS. Between Post Training and 24-h Post Training, AWS again showed a slightly steeper increase in NCS. This result contradicts our hypothesis of difficulties in motor sequence learning in AWS. In our study, apparently the amount of training, i.e., 160 repetitions of the sequence, was sufficient for AWS to catch up to ANS. This is in line with other studies that report that AWS can perform at a comparable level as ANS after a substantial amount of repetitions (Smits-Bandstra and Gracco, 2013, 2015). A larger number of repetitions is needed by AWS to catch up to the performance of ANS (Cooper and Allen, 1977 cited in Smits-Bandstra et al., 2006b).

To examine motor sequence learning in AWS and ANS without the influence of training, a separate analysis of only the Pre Training session was conducted. No learning or performance differences between ANS and AWS were detected, even though across all four blocks of Pre Training, AWS typed fewer correct sequences than ANS as can be seen in Figure 2. Both groups showed almost parallel motor learning for speed between the four blocks. Remarkable are the increases of NCS between blocks 1 and 2 in both groups. These may represent an intrinsic “motor adaptation as a setting up of a motor routine in a given novel setting” (Korman et al., 2003, p. 12496). A fast, finger motor adaptation in AWS is in contrast to the previously reported lower sensorimotor adaptation (Daliri and Max, 2018) or missing speech motor adaptation (Venkatagiri et al., 2013) in AWS.

#### The Number of Sequence Repetition

At first glance, the results of practice, retention, and early learning seem to contradict previous reports of a limited practice and retention abilities of AWS (e.g., Smits-Bandstra et al., 2006b; Sasisekaran and Weisberg, 2014; Bauerly and De Nil, 2015) or regular learning gains in speed (Webster, 1986; Bauerly and De Nil, 2011). However, we hold a similar view to Bauerly and De Nil (2015) in that the number of repetitions played a major role in our results, as averaging trials across entire blocks is reported to mask very early learning changes (Smits-Bandstra and Gracco, 2013). For example, within each block of the Pre Training session, the participants had already typed a large number of sequence repetitions (10–17 sequences per block). Most studies reporting lower motor performance gains in AWS during early learning compared only the first 5–9 sequence repetitions to the 25–30 sequence repetitions of 30 repetitions in total (Smits-Bandstra et al., 2006b Smith et al., 2010; Sasisekaran and Weisberg, 2014; Bauerly and De Nil, 2015). Studies using approximately 10–12 (Namasivayam and van Lieshout, 2008) or more than 30 sequence repetitions (e.g., Bauerly and De Nil, 2011) for average values could not replicate motor learning difficulties in kinematic measures to the same extent. For average values across 10–12 sequence repetitions, measuring parameters of fine movement coordination, however, distinguish motor sequence learning in AWS from ANS (Namasivayam and van Lieshout, 2008).

#### Task Complexity

The reduced task complexity in our experiment could have led us to miss differences in performance, adaptation, and early learning between the two groups. The complexity of the current five-item sequence “41324” may have been too low to detect learning deficits in AWS. However, depending on the number of sequence repetitions, more complex sequences can also fail to reveal learning differences between AWS and ANS (Bauerly and De Nil, 2011), and Webster (1986) succeeded in revealing motor practice differences for AWS using only a four-item finger tapping sequence. The finger tapping sequence in our present study consisted of five items with the repetition of one element. As the repetition of elements complicates sequence execution (Webster, 1986), this five-item sequence with repeated elements should have been more complex than those employed by Webster (1986). The key difference between previous results and ours is the number of sequence repetitions. Thus, the performance of AWS seems to be distinguishable from that of ANS, either if there are fewer than 10 sequence repetitions, or if task complexity increases, e.g., with longer sequences.

#### Task Duration

Yet, another explanation for the similarity between group performance could be due to the specific nature of the task: in previous studies, each sequence was typed after a “go” stimulus (Smits-Bandstra et al., 2006a, b; Bauerly and De Nil, 2015); in our experiment, participants typed the sequence repeatedly within 30-s intervals. It might be the case that participants developed a tapping rhythm while repeatedly typing the five-item sequence. Rhythmic movements are known to enhance motor performance (MacPherson and Collins, 2009). AWS benefit from external and internal rhythmic cues (e.g., metronome vs. finger tapping) such that following a metronome, finger tapping or singing lead to instant fluency (Bloodstein and Bernstein-Ratner, 2008). While AWS show synchronization difficulties to external auditory rhythms, they can keep self-paced rhythmic movements as stable as ANS (Falk et al., 2015; Hulstijn et al., 1992). Even though in our experiment, no external rhythmic stimulus was given, participants might have developed a rhythm by themselves. Rhythmic tapping would also explain that neither group showed a large increase of correct sequences at the first block of Post Training (Figure 2), indicating that Training may have not been as effective as reported by Korman et al. (2003). During Training, sequences were typed as response to a “go” stimulus preventing rhythmic tapping contrary to the 30-s intervals. We suggest that for participants who had already moved to a rhythmic tapping behavior during Pre Training, training the sequence individually had no further effect on movement patterns. This putative explanation remains speculative, however.

### Accuracy—TE

Error type enabled us to study the stability of cognitive motor sequence representations by differentiating within-sequence from between-sequence TE. The analysis of error type was based on the method of Albouy et al. (2012), who proposed that an increase of within-sequence TE might represent increasing variability of motor sequence execution.

After practice and retention, we reported a significant interaction between Group and Session for movement speed, revealing that during Pre Training, AWS typed fewer correct sequences than ANS but subsequently caught up in their performance. For accuracy, no interaction between Group and Session was observed, indicating that both groups showed similar motor sequence learning with regard to accuracy. Our results are in line with previous studies, reporting similar accuracy in both groups (e.g., Bauerly and De Nil, 2011, 2015; Sasisekaran and Weisberg, 2014). On the other side, AWS perform with lower accuracy when executing two tasks at the same time (Smits-Bandstra et al., 2006a; Smits-Bandstra and De Nil, 2009). Given that AWS seem to rely more on cognitive control for stable movement execution (Smits-Bandstra et al., 2006a), we assume that our study design (e.g., number of sequence repetitions or task duration) enabled AWS to perform the sequence without mental overload, such that enough capacity was left for controlling movement accuracy. This suggestion could also explain why descriptively AWS were even more accurate than ANS (Figure 3 and Table 4).

In our study, both groups made more within- than between-sequence TE. This difference was even larger in ANS than in AWS (comparing Pre, Post, and 24-h Post Training). Additionally, AWS made fewer TE than ANS comparing Pre Training to Transfer. Given the assumption that fewer within-sequence TE might represent a more stable representation of the sequence (Albouy et al., 2012), it seems that AWS were better in internalizing the practiced sequence. Models of motor sequence learning propose that the measure of accuracy represents progress within the visual–spatial component, which is more susceptible to explicit cognitive control (Penhune and Steele, 2012).

One could speculate that AWS were explicitly focusing on movement accuracy rather than speed. Attentional focus on accuracy leads to accurate but slow movements, whereas a focus on speed leads to fast but less accurate movements. This phenomenon is called speed–accuracy trade-off in motor performance (e.g., Fitts, 1954; Rival et al., 2003). Hence, different strategies of task execution might explain the group differences. However, two arguments speak against this suggestion. First, even though AWS gained more movement speed from Pre Training to Post Training than ANS, the pattern of accuracy remained similar across sessions, with AWS being more accurate than ANS at all times. Second, for skill acquisition, studies suggest that a focus on accuracy does not enhance motor learning (Solley, 1952; Lefebvre et al., 2012; Barnhoorn et al., 2019). Thus, we suggest that an attentional focus on accuracy in AWS would not have led to the current results of similar motor learning performance. Nevertheless, AWS may have benefited from a different interpretation of task instructions compared to ANS.

Socio-cognitive affective variables that lead to higher intrinsic motivation and attention can influence motor performance and motor learning (Wulf and Lewthwaite, 2016). For example, several studies have demonstrated that the type of received feedback (Saemi et al., 2012) or the focus of attention (Marchant et al., 2011; Wulf and Lewthwaite, 2016) affects motor learning. With reference to attention, this means that humans perform better when they focus on an external goal than when focusing on internal body states. For example, participants of a weightlifting experiment showed better performance when instructed with the external focus on “moving and exerting force through and against the barbell” than with the internal focus on “moving and exerting force with your arms” or the control condition “Perform as many repetitions as you can before failure” (Marchant et al., 2011, p. 468). During recruitment for this experiment, participants received the information that the study goal was to investigate motor learning in persons who stutter. Even though both groups volunteered for participation, persons who stutter were explicitly addressed as the target group. This may have raised an intrinsic motivation within many AWS to perform as well as they could, whereas control participants might not have felt the same urge for an outstanding performance. In addition, the goal, and with this the attentional focus, of AWS may have not been the one, which was introduced during the experiment, namely, “to type as fast and as accurately as possible” but to learn as well as they could.

### Relapse and Motor Sequence Learning

Limitations in motor sequence learning have been proposed as a possible factor for relapse after stuttering treatment programs including the acquisition and automatization of new speech techniques (Smits-Bandstra et al., 2006a, b; Smits-Bandstra and De Nil, 2009; Bauerly and De Nil, 2011). Psychological factors, such as attitude toward stuttering or speech, and the locus of control (i.e., the belief to what extent the outcome of events is controlled by oneself or by external forces) are known to increase the risk of relapse (Craig, 1998). However, these known factors could also influence motor sequence learning as socio-cognitive affective variables. In our study, after a training session and after overnight retention, we did not find poorer finger motor sequence learning in AWS than in ANS. Future studies addressing the link between relapse and motor sequence learning should try to encompass additional factors such as a longer pause between practice and generalization, automaticity levels, and socio-cognitive affective variables.

### Limitations of the Present Study

The argument of a higher intrinsic motivation and an external attentional focus in AWS remains only speculative, as we did not assess engagement or motivation in this study. Future studies investigating motor learning in persons who stutter should account for these socio-cognitive affective variables, such as intrinsic motivation, engagement, and attentional focus.

Because the results of motor sequence learning and performance across testing sessions did not confirm our hypotheses, we decided to add more fine-grained analyses: one *post hoc* analysis of early learning was conducted. As *post hoc* analysis increases the alpha error through multiple testing, the reported results must be regarded with caution. The robustness of our findings should be confirmed in future studies.

Variables such as the distinction between movement initiation and execution time (Webster and Ryan, 1991), reaction time for movement chunks (Smits-Bandstra and De Nil, 2013), or measures of movement coordination and stability (Namasivayam and van Lieshout, 2008) might have revealed more subtle differences within motor sequence learning and performance of AWS. In particular, further information about sequence chunking could reveal deeper insights into the underlying sequence representation.

## Conclusion

Adults who stutter succeeded in learning, retaining, and generalizing a five-item finger tapping sequence quantified by increased movement speed and accuracy as well as did ANS. Sufficient practice and the inclusion of a 24-h consolidation phase might have contributed to this outcome.

## Data Availability Statement

The datasets generated for this study are available on request to the corresponding author.

## Ethics Statement

The studies involving human participants were reviewed and approved by University Medical Center Göttingen ethics committee. The patients/participants provided their written informed consent to participate in this study.

## Author Contributions

AK, JC, and MS contributed to conception and design of the study. AK organized the database and wrote the first draft of the manuscript. AJ performed the statistical analysis. MH created the Perl scripts. All authors contributed to manuscript revision, and read, and approved the submitted version.

## Conflict of Interest

The authors declare that the research was conducted in the absence of any commercial or financial relationships that could be construed as a potential conflict of interest.

## Supplementary Material

FIGURE S1 Revised analysis of number of correct sequences: Practice, retention, and generalization with outliers excluded in panel **(A)** and outliers included in panel **(B)**. The mean of NCS is given for each block per test session. The blocks are not part of the conducted analyses, but are visualized for additional information of the participants’ learning progress. **(A)** Early learning, practice and retention effects on NCS for AWS (*n* = 14) and ANS (*n* = 15). The graph shows the analysis without the three outliers (error bars represent one standard deviation of intersubject variability unique to each group).

## Appendix

Here, we report the test statistics for the “Number of correct sequences: practice and retention” excluding the three outliers [two adults who stutter (AWS) and one ANS)]. The statistical procedure was the same as explained in section “Statistical Procedures.”

To analyze the effects of practice and retention, we included 6569 correct sequences into the generalized linear mixed-effects model with Poisson distribution. Similar results as in the original analysis (section “NCS: Practice and Retention”) were obtained. Both groups showed practice and learning effects, as the effect of Testing Session [χ^2^(2) = 139.4, *p* < 0.001] became significant. After Training, performance increased by approximately 30% [β = 0.3, SE = 0.03, 95% CI (0.24, 0.36)] on the first day. Overnight sleep additionally increased performance by 7% [β = 0.07, SE = 0.03, 95% CI (0.02, 0.12)]. The groups did not differ in performance [χ^2^(1) = 0.013, *p* = 0.72]. The interaction between Group and Testing Session was significant [χ^2^(2) = 6.19, *p* = 0.045]. *Post hoc* analyses were not significant but revealed that AWS typed fewer correct sequences during Pre Training but caught up to the performance of ANS at Post Training [β = 0.1, SE = 0.06, 95% CI (-0.02, 0.22)]. At 24-h Post Training, AWS typed even more sequences than ANS [β = 0.05, SE = 0.06, 95% CI (-0.06, 0.17)] (see Supplementary Figure 1A).

In addition, we analyzed the section “NCS: Generalization” including the three outliers. These results, however, should be interpreted with caution as systematic errors of the outliers lead to a reduced NCS within these participants.

The analysis of the ability to transfer the learned motor sequence (generalization) with the inclusion of outliers was based on 4268 correct sequences. As in the original results in section “NCS: Generalization,” both groups were able to generalize the learned sequence to the right hand as the significant effect of Testing Session indicates [χ^2^(1) = 54.6, *p* < 0.001]. No significant differences between Groups [χ^2^(1) < 1, *p* = 0.66] or Interaction of Group and Testing Session [χ^2^(1) = 2.7, *p* = 0.102] were detected (see Supplementary Figure 1B).
