# Supplementary figures and images for: Finger Sequence Learning in Adults Who Stutter
Source: Front Psychol. 2020 Jul 24;11:1543. doi: 10.3389/fpsyg.2020.01543 (PMC7396483; doi:10.3389/fpsyg.2020.01543)

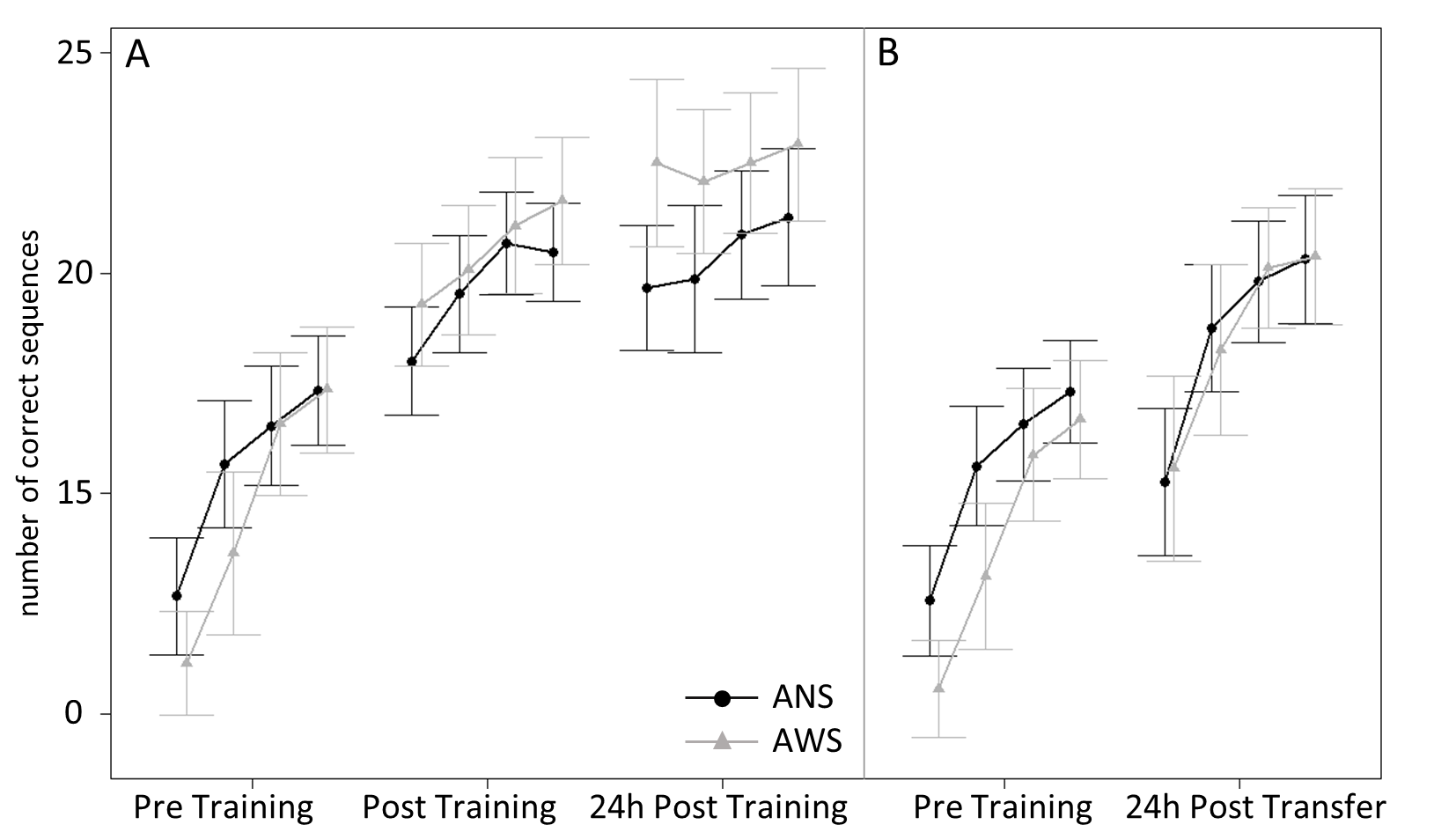

Supplement: FIGURE S1 — Revised analysis of number of correct sequences: Practice, retention, and generalization with outliers excluded in panel (A) and outliers included in panel (B). The mean of NCS is given for each block per test session. The blocks are not part of the conducted analyses, but are visualized for additional information of the participants’ learning progress. (A) Early learning, practice and retention effects on NCS for AWS (n = 14) and ANS (n = 15). The graph shows the analysis without the three outliers (error bars represent one standard deviation of intersubject variability unique to each group). [file Image_1.TIF]
